# Hybridization of the effective pharmacophores for treatment of epilepsy: design, synthesis, in vivo anticonvulsant activity, and in silico studies of phenoxyphenyl-1,3,4-oxadiazole-thio-*N*-phenylacetamid hybrids

**DOI:** 10.1186/s13065-023-01000-6

**Published:** 2023-07-17

**Authors:** Azadeh Fakhrioliaei, Fahimeh Abedinifar, Pedram Salehi Darjani, Maryam Mohammadi-Khanaposhtani, Bagher Larijani, Nematollah Ahangar, Mohammad Mahdavi

**Affiliations:** 1 Endocrinology and Metabolism Research Center, Endocrinology and Metabolism Clinical Sciences Institute, Tehran University of Medical Sciences, Tehran, Iran; 2 Student Researches Committee, Guilan University of Medical Sciences, Rasht, Iran; 3 Mobility Impairment Research Center, Health Institute, Babol University of Medical Sciences, Babol, Iran; 4 Cellular & Molecular Research Center, School of Medicine, Guilan University of Medical Sciences, Rasht, Iran; 5 Department of Pharmacology, School of Medicine, Guilan University of Medical Sciences, Rasht, Iran

**Keywords:** Anticonvulsant, 1,3,4-Oxadiazole, Phenoxyphenyl, Thio-*N*-phenylacetamid

## Abstract

**Background:**

Epilepsy is a common neurological disorder. The available drugs for this disease only control convulsions in nearly 70% of patients, while bearing many side effects. In this study, a new series of phenoxyphenyl-1,3,4-oxadiazole-thio-*N*-phenylacetamid hybrids **8a-m** was designed, synthesized, and evaluated as potent anticonvulsant agents.

**Methods:**

Phenoxyphenyl-1,3,4-oxadiazole-thio-*N*-phenylacetamid derivatives **8a-m** were synthesized with well-known chemical reactions and anticonvulsant activity of them was determined by pentylenetetrazole (PTZ) and maximal electroshock (MES) induced seizures in mice. Phenoxyphenyl-1,3,4-oxadiazole-thio-*N*-phenylacetamid scaffold has the necessary pharmacophores to be a benzodiazepine (BZD) receptor agonist, thus, the most potent anticonvulsant compounds were assayed in vivo and in silico as BZD receptor agonist. Furthermore, in vivo neurotoxicity evaluation and in silico physicochemical, pharmacokinetic, and toxicity study on the most potent compounds were also performed.

**Results:**

Obtained results demonstrated that two compounds among the title new compounds have anticonvulsant activity in PTZ test while all of the new compounds are active in the MES test. The best anticonvulsant activities were obtained with nitro derivatives **8k** and **8L**. In vivo evaluation of flumazenil effect (a BZD receptor antagonist) on anticonvulsant activity of compound **8k** confirmed that this compound is a BZD receptor agonist. The most potent compounds **8k** and **8L** interacted with the important residues of BZD-binding site of GABA_A_ receptor. Furthermore, neurotoxicity of the latter compounds was lower than positive control diazepam.

**Conclusion:**

According to these results, our designed scaffold can be a valuable lead structure for further structural developments and assessments to obtain a new potent anticonvulsant agent.

**Supplementary Information:**

The online version contains supplementary material available at 10.1186/s13065-023-01000-6.

## Introduction

Epilepsy is an important neurological disorder that affects nearly 50 million people worldwide [1]. Almost 90% of the epileptic patients live in developing countries [2]. This disease is characterized by the excessive temporal neuronal discharges that lead to uncontrolled convulsions [3]. The commercially available anticonvulsant medications show numerous side effects such as gingival hyperplasia, rash, ataxia, hepatotoxicity, vertigo, and megaloblastic anemia [4]. Furthermore, in one third of the epileptic patients, anticonvulsant drugs do not provide complete relief or control of seizures [5]. According to these points, there is high demand to develop effective and reliable agents for the treatment of epilepsy.

A useful tool for the design of new drugs in medicinal chemistry is molecular hybridization [6]. The basis of this method is to find effective pharmacophores from bioactive compounds and connecting them to each other in order to obtain a new lead compound for drug discovery.

**Fig. 1:**
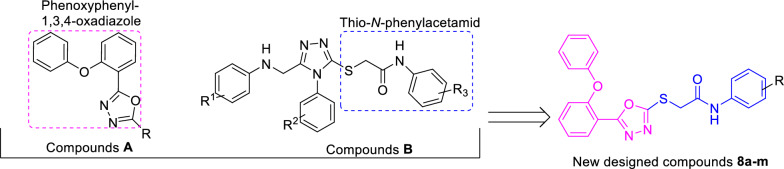
Use of molecular hybridization for design of the target compounds

One of the popular cores in the design of new anticonvulsant agents is 1,3,4-oxadiazole ring. Anticonvulsant potential of this ring has reported by various research groups [7–9]. Furthermore, 1,3,4-oxadiazole ring in combination with phenoxyphenyl group was found in the several series of the potent anticonvulsant agents such as compounds **A** (Fig. 1) [10]. On the other hand, Saidov et al. reported the synthesis and anticonvulsant activity of a series of thio-*N*-phenylacetamid derivatives **B** (Fig. 1) [11]. Consequently, our research group designed the new structures 8a-m by considering phenoxyphenyl-1,3,4-oxadiazole and thio-*N*-phenylacetamid of anticonvulsant agents **A** and **B** (Fig. 1). Designed phenoxyphenyl-1,3,4-oxadiazole-thio-*N*-phenylacetamid derivatives **8a-m** synthesized by simple chemical reactions, and their anticonvulsant and pharmacokinetic properties were evaluated by in vivo and in silico methods.

**Fig. 2:**
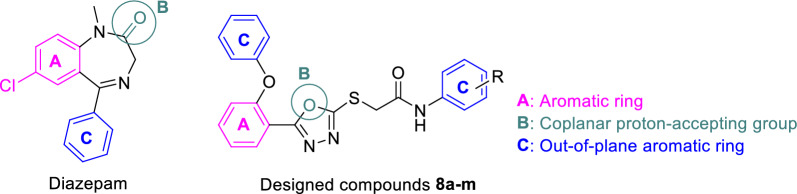
Structural characteristics of BZD receptor agonists: Diazepam (standard agonist) and newly designed compounds **8a–m**

Benzodiazepines (BZD) are an important class of anticonvulsant drugs [12]. These compounds are BZD receptor agonist, and in addition to epilepsy, they possess a known position in the treatment of various neurologic and psychiatric conditions like anxiety, alcohol withdrawal syndrome, insomnia, and muscle spasms [13]. BZDs exert the mentioned activities by positive allosteric effect on GABA_A_ receptors. The latter receptors are chloride ion channels, with the protein structure composing five subunits: one γ-, two β- and two α-subunits. BZDs *via* BZD binding site attach to GABA_A_ receptors. The key residues in BZD binding site are α1 Tyr159, α1 Tyr 209, α1 Thr206, α1 Vall211, α1 His101, and γ2 Phe77 [14]. The Structure Activity Relationship (SAR) of BZDs has revealed that a compound with the following structural properties could be a BZD receptor agonist: (A) an aromatic ring, (B) a co-planar proton accepting moiety in a suitable distance, and (C) second out-of‐plane aromatic ring (Fig. 2) [15]. Examining the structure of the newly designed compounds showed that these compounds have the necessary characteristics to be considered as BZD receptor agonists. Furthermore, to determine the mechanism of action of these new chemicals, in vivo and in silico studies were also performed.

## Result and discussion

### Chemistry

**Scheme 1:**
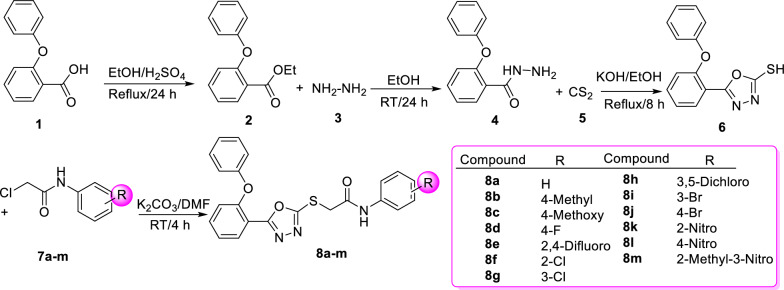
Synthetic strategy for the synthesis of phenoxyphenyl-1,3,4-oxadiazole-thio-*N*-phenylacetamid derivatives **8a-m**

The title compounds **8a-m** were prepared using the synthetic strategy described in Scheme 1. The first step of the synthetic method comprised the synthesis of ethyl 2-phenoxybenzoate **2** by esterification of 2-phenoxybenzoic acid **1** in the presences of ethanol and sulfuric acid. In the next step, 2-phenoxybenzoate **2** was reacted with hydrazine **3** to give 2-phenoxybenzohydrazide **4**. In the third step, the latter compound was converted to 5-(2-phenoxyphenyl)-1,3,4-oxadiazole-2-thiol **6** in the presence of carbon disulfide **5** in alcoholic potassium hydroxide [11]. In the final step, phenoxyphenyl-1,3,4-oxadiazole-thio-*N*-phenylacetamid derivatives **8a-m** were synthesized by reaction between compound **6** with *N*-phenyl-2-chloroacetamides **7a-m** in the presence of K_2_CO_3_ in DMF.

### Anticonvulsant activity

Newly synthesized compounds **8a-m** were evaluated for their in vivo anticonvulsant activities by the two recognized methods including PTZ and MES in mice [16]. The obtained results compared with diazepam as the positive control and demonstrated in Table 1. As can be seen in Scheme 1, in order to delineate the SAR and to get an optimized anticonvulsant agent, substituents on phenyl ring of thio-*N*-phenylacetamid moiety were altered.

**Table 1:** Anticonvulsant activity of the new compounds **8a–m** in PTZ and MES tests

Compound	Dose (mg/kg)	PTZ^a^		MES^b^	
		Number of animals protected / Number of tested animals	%Protection	Number of animals protected / Number of tested animals	%Protection
**8a**	1	–	–	5/8	62.5
	2	–	–	2/4	50
	5	0/4	0	2/4	50
	10	–	–	2/4	50
	20	–	–	2/4	50
**8b**	1	–	–	0/4	0
	10	–	–	1/4	25
	50	0/4	0	4/10	40
	100	–	–	3/4	75
**8c**	1	–	–	0/4	0
	2	0/4	0	1/4	25
	5	–	–	1/4	25
	10	–	–	1/4	25
	20	–	–	3/4	75
**8d**	1	–	–	1/4	25
	2	–	–	2/4	50
	5	0/4	0	2/4	50
	10	–	–	2/4	50
	20	–	–	1/4	25
**8e**	1	–	–	1/4	25
	5	0/4	0	1/4	25
	10	–	–	1/4	25
**8f**	5	0/4	0	0/4	0
	10	–	–	1/4	25
	20	–	–	3/4	75
**8g**	1	–	–	5/8	62.5
	2	–	–	2/8	25
	5	0/4	0	2/4	50
	10	–	–	3/4	75
	20	–	–	5/8	62.5
**8h**	1	–	–	0/4	0
	2	–	–	1/4	25
	5	0/4	0	1/4	25
	10	–	–	1/4	25
	20	–	–	2/4	50
**8i**	1	–	–	0/4	0
	5	0/4	0	2/4	50
	10	–	–	1/4	25
	20	–	–	2/4	50
**8j**	1	–	–	1/4	25
	5	0/4	0	2/4	50
	10	1/4	25	2/4	50
**8k**	2	0/4	0	3/4	75
	5	–	–	3/4	75
	10	–	–	2/4	50
**8k**^c^	2	–	–	0/4	0
**8L**	1	–	–	1/4	25
	2	0/4	0	1/4	25
	5	1/4	25	3/4	75
	10	0/4	0	2/4	50
**8m**	1	0/4	0	1/4	25
	2	–	–	2/4	50
	10	–	–	0/8	0
	50	–	–	0/4	0
Diazepam	2	6/6	100	6/6	100
Diazepam^c^	2	0/6	0	0/6	0

^a^Pentylentetrazole (100 mg/kg, ip) induced lethal convulsion

^b^Maximal electroshock seizure test: 50 mA, 60 Hz, ac, 0.2 s

^c^Flumazenil as a selective benzodiazepine receptor antagonist (1 mg/kg, ip) was administered 15 min before seizure induction

### Anticonvulsant activity against PTZ-induced seizure

As can be seen in Table 1, synthesized compounds **8a-m** in comparison to diazepam do not exhibit considerable anticonvulsant activity at the PTZ-induced convulsion assay. Among these compounds, compounds **8j** and **8L** exhibited a moderate activity with 25% protection at the doses 10 and 5 mg/kg, respectively.

### Anticonvulsant activity against MES-induced seizure

As is presented in Table 1, the newly synthesized compounds showed better protective profile against convulsion induced by MES in comparison to PTZ assay. The highest percentage of protection against induced seizure in MES assay was 75% that was obtained with compounds **8b**, **8c, 8f**, **8g**, **8k**, and **8L** at doses of 100, 20, 20, 10, 2, and 5 mg/kg, respectively. According to these results, the most potent anticonvulsant agent among the latter compounds is compound **8k** with 75% protection in the dose of 2 mg/kg. It should be noted that compound **8k**, in addition to the latter dose, showed 75% protection at dose of 5 mg/kg. In this series of compounds, the second potent compound was compound **8L** with 75% protection in the dose of 5 mg/kg.

### SAR study

The best compound in the PTZ test was 4-nitro derivative **8L**. Replacement of nitro substituent of compound **8L** with bromine atom, as in the case of compound **8j**, diminished the anticonvulsant activity while other substituents resulted in abolishment of the activity (Table 1). All new compounds were active in the MES test; the 2-nitro derivative **8k** and 4-nitro derivative **8L** showed the best profile. Replacement of 2-nitro substituent of compound **8k** with 2-chloro substituent, as compounds **8f**, led to a moderate decrease in anticonvulsant activity. On the other hand, changing the position of chlorine atom of 2-position to 3-position, in the case of compound **8g** (the third most potent anticonvulsant agent), led to a moderate increase in anticonvulsant activity. Moreover, 2-chloro analog **8f** and 4-methoxy analog **8c** had similar activity in the MES test. Introduction of methyl substituent instead of methoxy substituent led to a significant decrease in anticonvulsant activity (compound **8c** vs. compound **8b**). The rest of derivatives did not show significant anticonvulsant activity in the MES test.

### Comparison of the anticonvulsant activity of new compounds with the used patterns for their design

In this part, we compared the anticonvulsant activity of the newly synthesized compounds with the template compounds **A** and **B**.

**Scheme 2:**
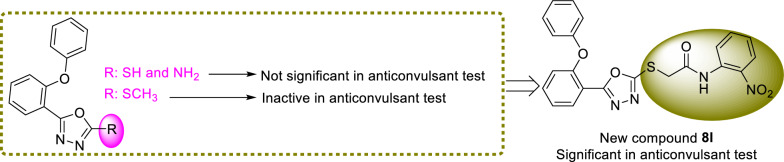
Comparison of anticonvulsant activity of template compounds **A** with the new compound **8l**

The comparison of anticonvulsant activity of the potent new compound **8L** with related analog of template compounds **A** revealed that adding of 2-nitro-*N*-phenylacetamid moiety dramatically increased anticonvulsant activity (Scheme 2) [10].

**Scheme 3:**
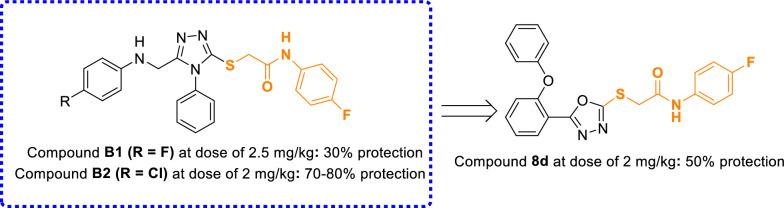
Comparison of percentage of protection in the anticonvulsant assay of new compound **8d** with its related analogs of template compounds **B**

Survey on the structures of template compounds **B** with the newly synthesized compounds **8** showed that two derivatives of compounds **B**, compound **B1** and compound **B2**, can be considered as corresponding analogs for new derivative **8d** (Scheme 3) [11]. Comparison of the percentage of protection in the anticonvulsant assay demonstrated that the new compound **8d** acted stronger than compound **B1** and weaker than compound **B2**.

### Evaluation of flumazenil effect on the anticonvulsant activity of compound 8k

To determine the mechanism of action of the new title compounds, effect of flumazenil as a BZD receptor antagonist on anticonvulsant activity of the compound **8k** as the most potent agent was evaluated in MES test. As can be observed in the Table 1, flumazenil antagonized anticonvulsant activity of the compound **8k** in the MES test. As a result, the involvement of BZD receptor in the anticonvulsant activity of the compound **8k** was confirmed.

### In vivo evaluation of neurotoxicity

The last in vivo assay was the determination of neurotoxic potential (muscle relaxant activity) of the potent anticonvulsant compounds **8k** and **8L** by rotarod method [17]. In this assay, the ability of animals pre-treated with these compounds was assessed in maintaining their balance on a rotating rod, and the results were compared to diazepam as the positive and DMSO as the negative control. As shown in Table 2, compounds **8k** and **8L** at the effective anticonvulsant doses exhibited less neurological deficit than diazepam.

**Table 2:** Muscle relaxant activity of selected compounds in rotarod test

Compound	Dose (mg/kg)	Time as seconds to stay on rotating bar
**8k**	2 mg/kg	15.98 ± 2.00*
**8L**	5 mg/kg	33.47 ± 5.75^ns^
DMSO	5 ml/kg	34.4 ± 4.48
Diazepam	2 mg/kg	3.80 ± 0.40***

Data is shown as Mean ± SEM. Comparing between different groups was conducted by ANOVA followed by Dunnett’s post-test. *ns* not significant *P ≤ 0.05, *** *P ≤ 0.001* compared to the DMSO group. (n = 4–6)

### Docking study

**Fig. 3:**
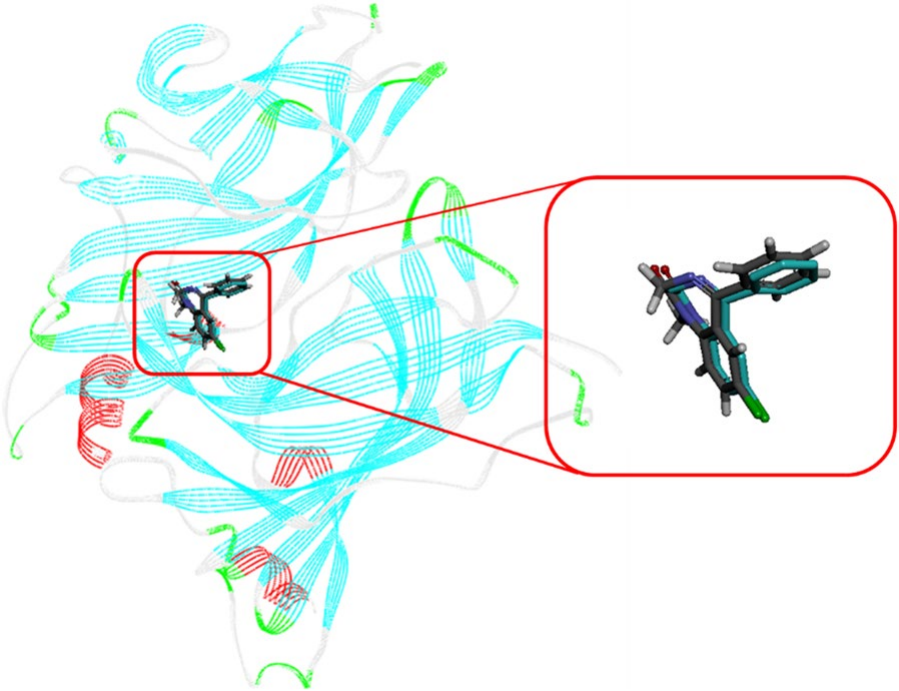
Structure of co-diazepam (gray) and re-docked diazepam (cyan) in the modeled BZD pocket

In addition to in vivo evaluation, an in-silico study was also performed to confirm the involvement of BZD receptor in the observed anticonvulsant activity of the newly synthesized compounds. In the first step of the docking study, validation method was performed and the co-inhibitor (diazepam) inside modeled BZD binding pocket was re-docked at its binding pocket (Fig. 3) [14]. The low root mean square deviation (RMSD) value for the re-docked complex with diazepam was 1.23 Å. Therefore, a valid performance was observed.

**Fig. 4:**
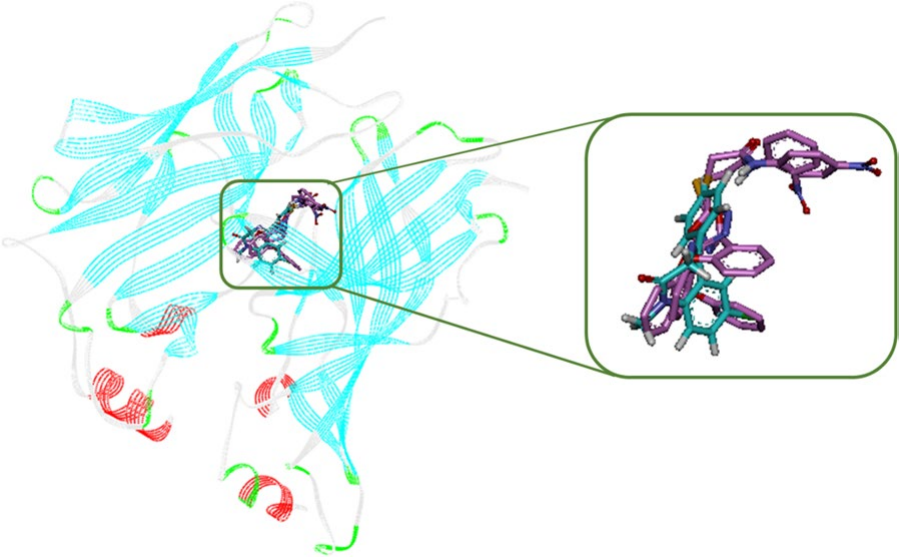
Diazepam (cyan) and the most potent compounds **8k** (pink) and **8l** (pink) superimposed in the BZD binding pocket of GABA_A_ receptor

Superimpose structure of diazepam and the selected compounds **8k** and **8L** in the BZD binding pocket of GABA_A_ receptor is shown in Fig. 4.

**Fig. 5:**
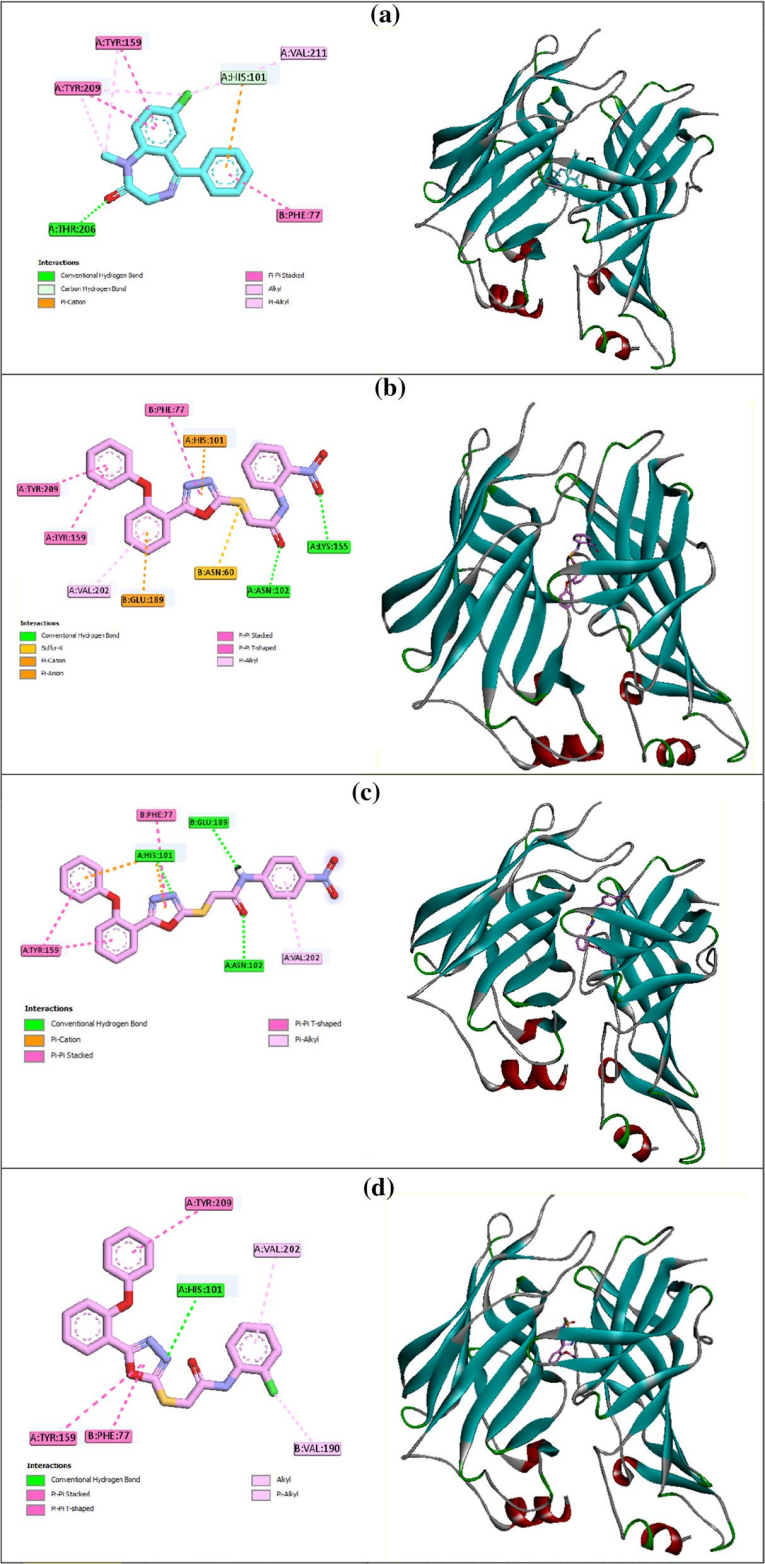
2D and 3D interaction modes of diazepam (**a**) and the most potent compounds **8k** (**b**), **8l** (**c**) and **8f** (**d**) in the BZD binding pocket of GABA_A_ receptor

Interaction modes of diazepam and the selected compounds are shown in Fig. 5. As can be seen in Fig. 5a, benzodiazepine moiety of diazepam established interactions with Tyr159 (π-π), Tyr 209 (π-π), Thr206 (hydrogen bond), and Vall211 (hydrophobic interaction). Furthermore, pendant phenyl ring of diazepam formed π-π and π-anion interactions with His101 and Phe77.

The most potent anticonvulsant agent **8k** established two hydrogen bonds with Lys155 and Asn102 *via* 2-nitro substituent and carbonyl unit, respectively (Fig. 5b). Sulfur atom of the compound **8k** interacted with residue Asn60 and 1,3,4-oxadiazole ring of this compound formed a π-cation interaction with His101 and a π-π interaction with Phe77. Phenoxyphenyl moiety of compound **8k** established the following interactions in the BZD binding pocket of GABA_A_ receptor: a π-anion interaction with Glu189, two π-π interactions with residues Tyr209 and Tyr159, and a hydrophobic interaction withVal202. Binding energy (BE) of compound **8k** was − 8.97 Kcal/mol.

The second potent anticonvulsant agent **8L** established three hydrogen bonds with BZD binding pocket *via* amide group (two interaction with Glu189 and Asn102) and 1,3,4-oxadiazole ring (an interaction with His101) (Fig. 5c). 1,3,4-Oxadiazole ring also formed a π-π interactions Phe77 and a π-cation interaction with His101. The latter amino acid also created another π-cation interaction with phenoxyphenyl moiety. Furthermore, compound **8L** established hydrophobic interactions with residues Tyr159 and Val202. BE value of compound **8L** was − 8.72 Kcal/mol.

In order to do more evaluation on the structure-activity relationships, docking study of 2-chloro derivative **8f** as a moderate anticonvulsant compound was performed (Fig. 5d). Interaction mode of compound 8f demonstrated this compound could form a hydrogen bond with His101 and several hydrophobic interactions with Val190, Phe77, Val202, Tyr159, and Tyr209. BE of this compound was − 7.5 Kcal/mol.

Survey on BEs of the studied compounds showed that the most potent compound **8k** had a lower free BE than the second potent compound **8L** and moderate compound **8f**, and therefore could easily bind to BZD binding pocket.

### Physicochemical properties and prediction of pharmacokinetic parameters and toxicity

Physicochemical properties of diazepam, compound **8k**, and compound **8L** were calculated by SwissADME online server [18]. Like diazepam, new compounds **8k** and **8L** followed of “Rule of Five” and are drug-likeness (Table 3). As can be seen in Table 3, these new compounds exhibited a negligible deviation from Veber Rule and were expected to have good human intestinal absorption (HIA).

**Table 3:** Physicochemical properties of diazepam, compound **8k**, and compound **8L**

Entry	MW	Clog P	HBD	HBA	RBC	tPSA
Rule of five	< 500	< 5	< 5	< 10	< 10	–
Veber rule	–	–	–	–	–	< 140
Diazepam	284.74	2.97	0	2	1	32.67
Compound **8k**	448.45	3.65	1	7	9	148.37
Compound **8L**	448.45	3.65	1	7	9	148.37

Pharmacokinetics and toxicity of diazepam and the most potent new anticonvulsants **8k** and **8L** were predicted by PreADMET and cbligand online softwares, and the obtained results were listed in Table 4 [19]. Diazepam had moderate permeability to Caco-2 cells while compounds **8k** and **8L** had poor permeability to these cells. Diazepam, compounds **8k** and **8L** had high HIA, and their permeability to the blood brain barrier (BBB score) is in the acceptable range [20]. In silico toxicity study demonstrated that all studied compounds are mutagen. This study also predicted that diazepam, compound **8k**, and compound **8L** did not possess carcinogenic effect on mouse and rat. Moreover, in term of cardiotoxicity (hERG inhibition), diazepam had medium risk while the new compounds **8k** and **8L** had low risk (Table 4).

**Table 4:** Pharmacokinetic and toxicity prediction of diazepam, compound **8k**, and compound **8L**

Druglikeness/ADME/T^a^	Compound		
	Diazepam	**8k**〹	**8L**〹
Rule of five	Suitable	Suitable	Suitable
Caco-2	47.6856	1.16966	1.62582
HIA	99.498312	96.206353	96.206446
BBB score	0.125	0.038	0.038
Ames test	Mutagen	Mutagen	Mutagen
Carcino mouse	Negative	Negative	Negative
Carcino rat	Negative	Negative	Negative
hERG inhibition	Medium risk	Low risk	Low risk

^a^The recommended ranges for Caco2: <25 poor, > 500 great, HIA: >80% is high < 25% is poor, BBB score > 0.02, and Skin_Permeability = − 8.0 – − 1.0.

## Conclusion

In this study a new series of phenoxyphenyl-1,3,4-oxadiazole-thio-*N*-phenylacetamid derivatives **8a-m** designed as potent anticonvulsant agents. These compounds synthesized *via* simple and efficient chemical reactions. All synthesized chemicals showed anticonvulsant activity in the MES test while with the exception of two compounds, the rest of derivatives were inactive in PTZ test. Among the synthesized compounds, the most potent anticonvulsant compounds were **8k** and **8L** with 75% protection in doses 2 and 5 mg/kg, respectively. Compounds **8k** and **8L** also showed by far more acceptable neurotoxic effect in comparison to diazepam. Anticonvulsant activity of the compound **8k** was inhibited by flumazenil as a BZD receptor antagonist. On the other hand, this compound interacted with important residues of BZD-binding site of the GABA_A_ receptor. As a consequence, compound **8k** can be a BZD receptor agonist. In silico pharmacokinetic studies predicted that the selected compounds **8k** and **8L** possess satisfactory features as a drug candidate.

## Experimental

### Synthesis of ethyl 2-phenoxybenzoate 2

2-Phenoxybenzoic acid **1** (20 mmol) in the presences of sulfuric acid (1 ml) in ethanol (50 ml) was stirred at reflux condition for 24 h. Then, the solvent was evaporated under reduced pressure, the obtained residue was dissolved in ethyl acetate (25 ml), and the organic phase was washed with water (3 × 20 ml) and brine (25 ml). The ethyl acetate phase was dried by Na_2_SO_4_ and the ethyl acetate was evaporated to give pure 2-phenoxybenzoate **2**.

### Synthesis of 2-phenoxybenzohydrazide 4

A mixture of 2-phenoxybenzoate **2** (20 mmol) and hydrazine **3** (20 mmol) in ethanol (50 ml) was stirred at room temperature for 24 h. After that, water (40 ml) was added to the reaction mixture and formed participate was filtered and dried at 60 °C to obtain pure 2-phenoxybenzohydrazide **4**.

### Synthesis of 5-(2-phenoxyphenyl)-1,3,4-oxadiazole-2-thiol 6

2-Phenoxybenzohydrazide **4** (20 mmol) and carbon disulfide **5** (20 mmol) were added to a mixture of potassium hydroxide (20 mmol) in EtOH (50 ml) at room temperature. Then, this mixture was heated under reflux for 8 h. After completion of the reaction (checked by TLC), water was added to the reaction mixture and the final mixture was filtrated to obtain pure 5-(2-phenoxyphenyl)-1,3,4-oxadiazole-2-thiol **6**.

### General synthesis of phenoxyphenyl-1,3,4-oxadiazole-N-phenylacetamid derivatives 8a-m

A suspension of 5-(2-phenoxyphenyl)-1,3,4-oxadiazole-2-thiol **6** (1 mmol), *N*-phenyl-2-chloroacetamide derivatives **7a-m** (1 mmol), and potassium carbonate (1.2 mmol) in DMF (5 ml) was stirred at room temperature for 4 h. Thereafter, water was added to the reaction mixture and the obtained participates were filtrated and recrystallized in ethyl acetate to give target compounds **8a-m** (Additional file 1).

#### 2-((5-(2-phenoxyphenyl)-1,3,4-oxadiazol-2-yl)thio)- *N* -phenylacetamide (8a)

White solid, Yield: 69%, m.p.: 176–178 °C; IR (KBr) ν (cm^–1^): 3102, 3084, 1680. ^1^ H NMR (DMSO-*d*_6_) δ (ppm) (300 MHz): 4.32 (2 H, s, CH_2_), 7.00 (2 H, d, *J* = 9 Hz, H2’’, H6’’), 7.07–7.17 (3 H, m, H4, H5’, H4’’), 7.32–7.47 (5 H, m, H3’, H5, H3, H3’’, H5’’), 7.59–7.65 (3 H, m, H4’, H2,6), 7.98–8.01(1 H, dd, *J* = 9,3 Hz, H2’), 10.4 (1 H, s, NH). ^13^ C NMR (125 MHz, DMSO-*d*_6_), δ ppm: 31.1, 37.2, 115.5, 118.7, 119.6, 120.6, 124.1, 124.2, 124.7, 129.3, 130.5, 130.6, 134.1, 139.1, 154.6, 156.8, 163.5, 164.0, 165.2. Anal. Calcd for C_22_H_17_N_3_O_3_S: C, 65.49; H, 4.25; N, 10.42; Found C, 65.48; H, 4.26; N, 10.41.

#### 2-((5-(2-phenoxyphenyl)-1,3,4-oxadiazol-2-yl)thio)- *N* -(p-tolyl)acetamide (8b)

White powder, Yield: 71%, m.p.: 177–179 °C; IR (KBr) ν (cm^–1^): 3102, 3067, 1691. ^1^ H NMR (DMSO-*d*_6_) δ (ppm) (300 MHz): 2.27(3 H, s, CH_3_), 4.30 (2 H, s, CH_2_), 7.02 (2 H, d, *J* = 9 Hz, H2’’,H6’’), 7.08–7.18 (4 H, m, H3,H5, H3’, H4’’), 7.33–7.42 (3 H, m, H3’’,5’’, H4’), 7.48 (2 H, d, *J* = 9 Hz, H2,6), 7.60–7.66(1 H, m, H4’), 7.98-8.00 (1 H, dd, *J* = 9,3 Hz, H2’), 10.34 (1 H, s, NH). ^13^ C NMR (125 MHz, DMSO-*d*_6_), δ ppm: 20.9, 37.2, 115.5, 118.7, 119.6, 120.7, 124.1, 124.7, 129.6, 130.5, 130.6, 133.1, 134.1, 136.6, 154.6, 156.8, 163.5, 164.0, 164.9. Anal. Calcd for C_23_H_19_N_3_O_3_S: C, 66.17; H, 4.59; N, 10.07; Found C, 65.17; H, 4.63; N, 10.1.

#### *N*-(4-methoxyphenyl)-2-((5-(2-phenoxyphenyl)-1,3,4-oxadiazol-2-yl)thio)acetamide (8c)

White powder, Yield: 68%, m.p.: 173–175°C; IR (KBr) ν (cm^–1^): 3100, 3067, 1680. ^1^H NMR (DMSO-*d*_6_) δ (ppm) (300 MHz): 3.74(3H, s, CH3), 4.28 (2H, s, CH_2_), 6.92(2H, d, *J* = 9 Hz, H3,H5), 7.02 (2H, d, *J* = 9 Hz, H2’’,H6’’ ), 7.10 (1 H, d, *J* = 6 Hz, H5’), 7.13–7.18(1 H, t, *J* = 6 Hz, H4’’), 7.33–7.42 (3 H, m, H3’, H3’’, H5’’), 7.52 (2 H, d, *J* = 9 Hz, H2, H6), 7.60–7.66(1 H, m, H4’), 7.98-8.00 (1 H, dd, J = 6,3 Hz, H2’), 10.28 (1 H, s, NH). ^13^ C NMR (125 MHz, DMSO-*d*_6_), δ ppm: 37.1, 55.6, 114.4, 115.5, 118.7, 120.7, 121.2, 124.1, 124.7, 130.5, 130.6, 132.2, 134.1, 154.6, 155.9, 156.8, 163.5, 164.0, 164.6. Anal. Calcd for C_23_H_19_N_3_O_4_S: C, 63.73; H, 4.42; N, 9.69; Found C, 63.71; H, 4.43; N, 9.71.

#### *N*-(4-fluorophenyl)-2-((5-(2-phenoxyphenyl)-1,3,4-oxadiazol-2-yl)thio)acetamide (8d)

White powder, Yield: 72%, m.p.: 184–186 °C; IR (KBr) ν (cm^–1^): 3106, 3067, 1680. ^1^ H NMR (DMSO-*d*_6_) δ (ppm) (300 MHz): 4.30 (2 H, s, CH_2_), 7.00 (2 H, dd, *J* = 3,6 Hz, H2’’, H6’’), 7.10 (1 H, d, *J* = 9 Hz, H5’), 7.13–7.20(3 H, m, H3, H5, H4’’), 7.33–7.42 (3 H, m, H3’, H3’’, H5’’), 7.59–7.65(3 H, m, H2, H6, H4’), 7.96–8.01(1 H, m, H2’), 10.4 (1 H, s, NH). ^13^ C NMR (125 MHz, DMSO-*d*_6_), δ ppm: 37.0, 115.5, 115.7, 116.0, 118.7, 120.7, 121.4, 130.5, 130.6(d, J = 17), 134.1, 135.4(d, J = 5), 154.6, 156.8, 157.0, 160.2, 163.5, 163.9, 165.1. Anal. Calcd for C_22_H_16_FN_3_O_3_S: C, 62.78; H, 3.76; N, 9.97; Found C, 54.73; H, 3.43; N, 8.70.

#### *N*-(2,4-difluorophenyl)-2-((5-(2-phenoxyphenyl)-1,3,4-oxadiazol-2-yl)thio)acetamide (8e)

White powder, Yield: 67%, m.p.: 185–186 °C; IR (KBr) ν (cm^–1^): 3100, 3067, 1684. ^1^ H NMR (DMSO-*d*_6_) δ (ppm) (300 MHz): 4.37 (2 H, s, CH_2_), 7.02 (2 H, dd, *J* = 3,9 Hz,H3, 5), 7.10 (2 H, d, *J* = 9 Hz, H2’’,6’’), 7.15(1 H, m, H5’), 7.28–7.41 (4 H, m, H3’, H4’, H3’’, H5’’), 7.59–7.64(1 H, m, H4’’), 7.83–7.91(1 H, m, H6), 8.01(1 H, dd, *J* = 3,9 Hz, H2’), 10.2 (1 H, s, NH). ^13^ C NMR (125 MHz, DMSO-*d*_6_), δ ppm: 36.5, 104.3, 104.6(d, J = 5 Hz), 104.9, 111.4(d, J = 6.2 Hz), 111.7 (d, *J* = 6.2 Hz), 115.5, 118.7, 120.6, 122.8 (dd, *J* = 20, 6.2 Hz), 124.1, 124.7, 125.6(dd, *J* = 11.2, 6.2 Hz), 130.5(d, *J* = 15 Hz), 134.1, 152.4(d, *J* = 20), 154.6, 1557(d, *J* = 20), 156.8, 157.3(d, *J* = 20), 160.6(d, *J* = 18.7), 163.6, 163.8, 165.9. Anal. Calcd for C_22_H_15_F_2_N_3_O_3_S: C, 60.13; H, 3.44; N, 9.56; Found C, 59.73; H, 3.96; N, 9.54.

#### *N*-(2-chlorophenyl)-2-((5-(2-phenoxyphenyl)-1,3,4-oxadiazol-2-yl)thio)acetamide (8f)

White powder, Yield: 70%, m.p.: 183–185 °C; IR (KBr) ν (cm^–1^): 3100, 3047, 1685. ^1^ H NMR (DMSO-*d*_6_) δ (ppm) (300 MHz): 4.31 (2 H, d, *J* = 6 Hz, CH_2_-N), 7.01 (2 H, d, *J* = 9 Hz,H2’’,6’’), 7.09(1 H, d, *J* = 9 Hz, H4), 7.15(1 H, dd, J = 6,9 Hz, H4’’), 7.33–7.42(5 H, m, H3’,H5’,H5,H3,H4’), 7.62–7.65(3 H, m, H5’’, H3’’,H2’), 7.98–8.01(1 H, m, H6), 10.5 (1 H, m, NH). ^13^ C NMR (125 MHz, DMSO-*d*_6_), δ ppm: 37.1, 115.5 118.7, 120.6, 121.2, 124.1, 124.7, 127.7, 129.2, 130.5, 130.6, 134.1, 138.0, 154.6, 156.8, 163.5, 163.9, 165.3. Anal. Calcd for C_22_H_16_ClN_3_O_3_S: C, 60.34; H, 3.68; N, 9.60; Found C, 60.36; H, 3.69; N, 9.61.

#### *N*-(3-chlorophenyl)-2-((5-(2-phenoxyphenyl)-1,3,4-oxadiazol-2-yl)thio)acetamide (8g)

White powder, Yield: 66%, m.p.: 180–185 °C; IR (KBr) ν (cm^–1^): 3202, 3088, 1685. ^1^ H NMR (DMSO-*d*_6_) δ (ppm) (300 MHz): 4.31 (2 H, s, CH_2_), 7.00 (2 H, d, *J* = 9 Hz, H2’’,6’’), 7.07–7.18 (3 H, m, H4, H5’, H4’’), 7.31–7.42 (5 H, m, H3’, H5, H3, H3’’, H5’’), 7.59–7.63(1 H, m, H2’), 7.80(1 H, t, *J* = 3 Hz, H2), 7.98(1 H, dd, *J* = 3,6 Hz, H6 ) 10.6 (1 H, s, NH). ^13^ C NMR (125 MHz, DMSO-*d*_6_), δ ppm: 37.1, 115.5, 118.0, 118.7, 119.1, 120.6, 123.8, 124.1, 124.7, 130.5, 130.6, 131.0, 133.6, 134.1, 140.5, 154.6, 156.8, 163.6, 163.9, 165.5. Anal. Calcd for C_22_H_16_ClN_3_O_3_S: 60.34; H, 3.68; Cl, 8.10; N, 9.60; Found C, 60.35; H, 3.69; N, 9.58.

#### *N*-(3,5-dichlorophenyl)-2-((5-(2-phenoxyphenyl)-1,3,4-oxadiazol-2-yl)thio)acetamide (8h)

White powder, Yield: 68%, m.p.: 178–181°C; IR (KBr) ν (cm–1): 3110, 3070, 1684. ^1^H NMR (DMSO-*d*_6_) δ (ppm) (300 MHz): 4.31 (2H, s, CH_2_), 7.00 (2H, d, *J* = 9 Hz, H2’’,H6’’), 7.08 (1H, d, *J* = 9 Hz, H5’), 7.15 (1H, dd,, *J* = 9 ,6 Hz, H4’’ ), 7.29 (1H, dd, *J =* 2Hz, H4), 7.32–7.41(3H, m, H3’, H3’’, H5’’), 7.59–7.65(3H, m, H4’, H2, H6), 7.98(1H, dd, *J* = 3,6 Hz, H2’ ) 10.7 (1 H, s, NH). ^13^ C NMR (125 MHz, DMSO-*d*_6_), δ ppm: 37.1, 115.4, 117.7, 118.7, 120.6, 123.3, 124.3, 124.6, 130.5, 130.6, 134.1, 134.6, 141.3, 154.6, 156.8, 163.6, 163.8, 166.0. Anal. Calcd for C_22_H_15_Cl_2_N_3_O_3_S: C, 55.94; H, 3.20; N, 8.90; Found C, 55.93; H, 3.26; N, 8.84.

#### *N*-(3-bromophenyl)-2-((5-(2-phenoxyphenyl)-1,3,4-oxadiazol-2-yl)thio)acetamide (8i)

White powder, Yield: 72%, m.p.: 176–178 °C; IR (KBr) ν (cm^–1^): 3100, 3085, 1688. ^1^ H NMR (DMSO-*d*_6_) δ (ppm) (300 MHz): 4.31 (2 H, s, CH_2_), 7.00 (2 H, d, *J* = 6 Hz, H2’’, H6’’), 7.08 (1 H, d, *J* = 6 Hz, H5’), 7.15(1 H, t, *J* = 6 Hz, H4’’), 7.29–7.42 (5 H, m, H4, H4’, H5, H3’’,H5’’), 7.47–7.51(1 H, m, H2’), 7.60–7.63(1 H, m, H6), 7.9 (1 H, s, H2), 10.6 (1 H, s, NH). ^13^ C NMR (125 MHz, DMSO-*d*_6_), δ ppm: 37.1, 115.5, 118.4, 118.7, 120.6, 121.9, 124.1, 124.7, 126.7, 130.5, 130.6, 131.3, 134.1, 140.6, 154.6, 156.8, 163.6, 163.8, 165.6. Anal. Calcd for C_22_H_16_BrN_3_O_3_S: C, 54.78; H, 3.34; N, 8.71; Found C, 54.73; H, 3.43; N, 8.70.

#### *N*-(4-bromophenyl)-2-((5-(2-phenoxyphenyl)-1,3,4-oxadiazol-2-yl)thio)acetamide (8j)

White powder, Yield: 69%, m.p.: 185–187 °C; IR (KBr) ν (cm^–1^): 3102, 3067, 1690. ^1^ H NMR (DMSO-*d*_6_) δ (ppm) (300 MHz): 4.31 (2 H, s, CH_2_), 7.00 (2 H, d, *J* = 6 Hz, H2’’, H6’’), 7.10 (1 H, d, *J* = 6 Hz, H5’), 7.16(1 H, t, *J* = 6 Hz, H4’’), 7.38–7.42 (3 H, m, H3’, H3’’,H5’’), 7.50–7.60 (4 H, m, H2, H6, H2, H3, H5), 7.61–7.66(1 H, m, H4’), 7.96–8.01(1 H, m, H2’), 10.5 (1 H, s, NH). ^13^ C NMR (125 MHz, DMSO-*d*_6_), δ ppm: 37.1, 115.5, 115.7, 118.7, 120.7, 121.5, 124.1, 124.7, 130.5, 130.6, 130.9, 132.1, 132.5, 138.4, 154.6, 156.8, 163.5, 163.4, 165.6. Anal. Calcd for C_22_H_16_BrN_3_O_3_S: C, 54.78; H, 3.34; N, 8.71; Found C, 54.73; H, 3.43; N, 8.70.

#### *N*-(2-nitrophenyl)-2-((5-(2-phenoxyphenyl)-1,3,4-oxadiazol-2-yl)thio)acetamide (8k)

White powder, Yield: 73%, m.p.: 180–181°C; IR (KBr) ν (cm^–1^): 3102, 3064, 1686. ^1^H NMR (DMSO-*d*_6_) δ (ppm) (300 MHz): 4.34 (2H, s, CH_2_), 7.02 (2H, d, *J* = 9 Hz, H2’’,6’’), 7.10 (1H, d, *J* = 9 Hz, H5’), 7.16 (1H, dd, *J* = 6,9, H4’’), 7.33–7.42 (4H, m, H3’, H4, H3’’,H5’’ ), 7.61–7.67(1 H, m, H4’), 7.73–7.75(2 H, m, H5, H2’), 7.99–8.02 (2 H, m, H3, H6), 10.4 (1 H, s, NH). ^13^ C NMR (125 MHz, DMSO-*d*_6_), δ ppm: 36.5, 115.5, 118.7, 120.6, 124.1, 124.7, 125.5, 125.6, 126.1, 130.5, 130.7, 131.2, 134.2, 134.6, 142.5, 154.6, 156.8, 163.5, 163.6, 165.9. Anal. Calcd for C_22_H_16_N_4_O_5_S: C, 58.92; H, 3.60; N, 12.49; Found C, 58.73; H, 3.63; N, 12.5.

#### *N*-(4-nitrophenyl)-2-((5-(2-phenoxyphenyl)-1,3,4-oxadiazol-2-yl)thio)acetamide (8L)

White powder, Yield: 69%, m.p.: 183–185 °C; IR (KBr) ν (cm^–1^): 3102, 3066, 1675. ^1^ H NMR (DMSO-*d*_6_) δ (ppm) (300 MHz): 4.38 (2 H, s, CH_2_), 7.00 (2 H, d, *J* = 6 Hz, H2’’,H6’’), 7.08 (1 H, d, *J* = 6 Hz, H5’), 7.14 (1 H, dd, *J* = 6,9, H4’’), 7.33–7.41 (3 H, m, H3’,H5’’,H4’), 7.59–7.65(1 H, m, H3’’), 7.84(2 H, d, J = 9 Hz, H2, H6), 7.97-8.00 (1 H, m, H2’), 8.24(2 H, d, *J* = 9 Hz, H3,5), 11.0 (1 H, s, NH). ^13^ C NMR (125 MHz, DMSO-*d*_6_), δ ppm: 37.2, 115.4, 118.7, 119.3, 120.6, 124.1, 124.7, 125.5, 130.5, 130.6, 134.1, 142.9, 145.1, 154.6, 156.8, 163.6, 163.8, 166.3. Anal. Calcd for C_22_H_16_N_4_O_5_S: C, 58.92; H, 3.60; N, 12.49; Found C, 58.73; H, 3.63; N, 12.5.

#### *N*-(2-methyl-3-nitrophenyl)-2-((5-(2-phenoxyphenyl)-1,3,4-oxadiazol-2-yl)thio)acetamide (8m)

White powder, Yield: 70%, m.p.: 180–182°C; IR (KBr) ν (cm–1): 3100, 3064, 1680. ^1^H NMR (DMSO-*d*_6_) δ (ppm) (300 MHz): 2.25(3H, s, CH3), 4.37 (2H, s, CH_2_), 7.03 (2H, dd, *J* = 9,3 Hz, H2’’,H6’’ ), 7.11 (1 H, dd, *J* = 9,3 Hz, H5’), 7.14–7.19(1 H, m, H4’’), 7.35–7.44 (4 H, m, H2’, H4’, H3’’, H5’’), 7.61–7.69(2 H, m, H2’, H5), 7.76(1 H, dd, *J* = 9,3 Hz, H6), 8.02(1 H, dd, *J* = 3,6 Hz, H4), 10.2 (1 H, s, NH). ^13^ C NMR (125 MHz, DMSO-*d*_6_), δ ppm: 14.1, 36.4, 115.5, 118.7, 119.5, 119.7, 120.7, 121.5, 124.2, 124.7, 127.0, 127.3, 130.3, 130.5, 130.7, 134.2, 138.0, 151.3, 154.6, 156.8, 163.6, 163.9, 166.0. Anal. Calcd for C_23_H_18_N_4_O_5_S: C, 59.73; H, 3.92; N, 12.11; Found C, 59.73; H, 3.94; N, 12.10.

### Anticonvulsant activity

Male NMRI mice (20–30 g weight, 3 months old) were used for the assessment of anticonvulsant potential of the synthesized compounds. The animals were prepared by the Center for Breeding and Care of Laboratory animals, School of Pharmacy, Guilan University of Medical Sciences, Rasht, Iran. The animals were kept at 25 ± 2 °C, 12 h light/dark cycle in the standard Plexiglas cage with free access to food and water. Animals were transferred to the research laboratory at least 1 h before the experiments, and each animal was just used for one experiment. The synthesized chemicals were administered 30 min before the induction of convulsion by the MES (Maximal Electroshock) or PTZ (Pentylenetetrazol) method, and the number of protected animals against the induced convulsion was noted. To evaluate the possible role of benzodiazepine receptors in the effect of the most potent synthesized compounds, Flumazenil (1 mg/kg) was administered 15 min prior to seizure induction. In the MES test, a current with characteristics of 60 Hz and 50 mA was administered for 0.2 s via ear electrodes previously moistened with normal saline for more effective delivery of the current. The abolishment of the Hind Leg Tonic Extension (HLTE) was considered as the protection of animal against the convulsion. In the PTZ test, PTZ was injected in the dose of 100 mg/kg, the subjects were carefully observed for the next 0.5 h for the occurrence of the tonic-clonic lethal convulsion, and the results were represented as the number of protected animals to the number of tested animals [16].

The rout of administration for the different substances was intraperitoneal (i.p) injection. DMSO was used as the solvent for the preparation of fresh solution of flumazenil and the synthesized compounds; PTZ was dissolved in normal saline 0.9%. Final volume of injection for the solutions containing DMSO and normal saline 0.9% was 5 and 10 ml/kg body weight, respectively. Moreover, in both the PTZ and MES tests, diazepam (2 mg/kg), a benzodiazepine receptor agonist with prominent anticonvulsant properties was administered as the positive control, and a separate group of animals received DMSO as the negative control. The number of animals used for each dose/experiment was 4, and the numbers more than 4 illustrated in Table 1 for some chemicals indicates repeat of experiment for the clarification of results. At the end of experiments, CO2 was used to euthanize the animals.

### Rotarod test (acute neurotoxicity)

The most potent anticonvulsant agents **8k** and **8L**, diazepam as the positive control, and DMSO as the negative control were i.p administered to the mice, and their capabilities to stay on the rotating rod (5 rpm) was recorded 30 min following injections [17].

### Docking study

To investigate the interactions of the newly synthesized compounds within the BZD binding pocket, a docking study was conducted using AutoDock Tools (version 1.5.6). Since there was no crystallographic structure of GABA_A_ receptor in the protein data bank, we used Richter et al.‘s homology model for the BZD binding pocket. This model was a diazepam-bound GABA_A_ receptor with pdb format [14]. The 3D structure of the selected compounds **8k**, **8L**, and **8f** was provided using MarvineSketch 5.8.3, 2012, ChemAxon (http://www.chemaxon.com) and the obtained PDB formats were converted to PDBQT formats using AutoDock Tools version 1.5.6 (http://mgltools.scripps.edu). The same software also provided the PDBQT format of the BZD binding pocket. Docking grid box for this study was placed in 40 × 40 × 40 Å points with x = 43.640, y = 43.866 and z = 9.3290 Å directions and each docked system was carried out by 50 runs (AUTODOCK search, Lamarckian genetic algorithm). The obtained ligand-receptor complex conformations were evaluated by BIOVIA Discovery Studio v.3.5.

### Physicochemical properties and prediction of ADMET

The physicochemical properties of the selected compounds **8k** and **8L** and the positive control diazepam were calculated by SwissADME online server (http://www.swissadme.ch/) [18]. ADME and toxicity profile of the latter compounds were predicted by PreADMET web servers (https://preadmet.bmdrc.kr/) [19]. BBB penetration of the selected compounds and positive control was predicted by online BBB predictor (www.cbligand.org) [20].

## Supplementary Information

**Additional file 1.** Support information.

## Data Availability

The datasets used or analyzed during the current study are available from the corresponding authors on reasonable request.
