# Supplementary material for: Hybridization of the effective pharmacophores for treatment of epilepsy: design, synthesis, in vivo anticonvulsant activity, and in silico studies of phenoxyphenyl-1,3,4-oxadiazole-thio-N-phenylacetamid hybrids
Source: BMC Chem. 2023 Jul 17;17(1):80. doi: 10.1186/s13065-023-01000-6 (PMC10353189; doi:10.1186/s13065-023-01000-6)
Supplement: Supplementary file 1 — Additional file 1. Support information. [file 13065_2023_1000_MOESM1_ESM.docx]

**Support information**

*2-((5-(2-phenoxyphenyl)-1,3,4-oxadiazol-2-yl)thio)-N-phenylacetamide (****8a****)*

*
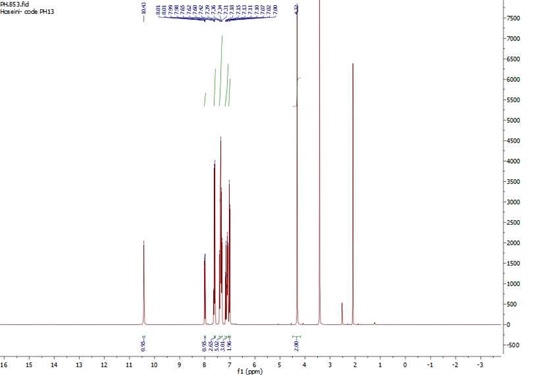
*

*
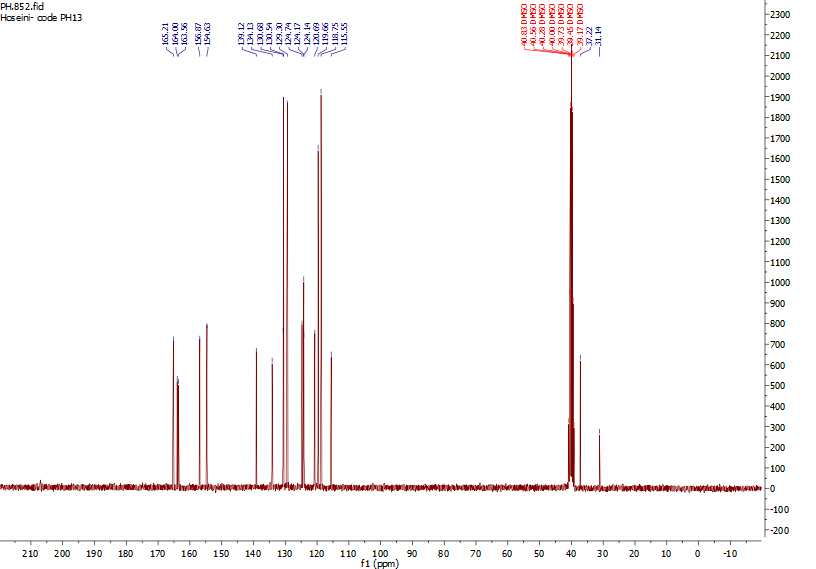
*

*2-((5-(2-phenoxyphenyl)-1,3,4-oxadiazol-2-yl)thio)-N-(p-tolyl)acetamide (****8b****)*

*
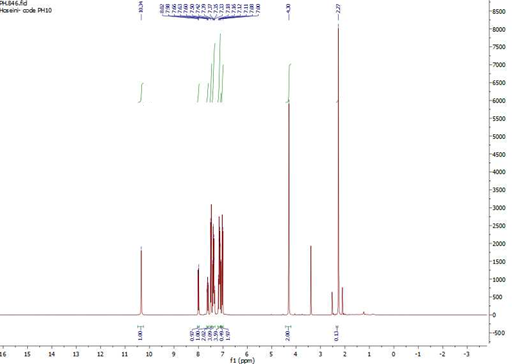
*

*
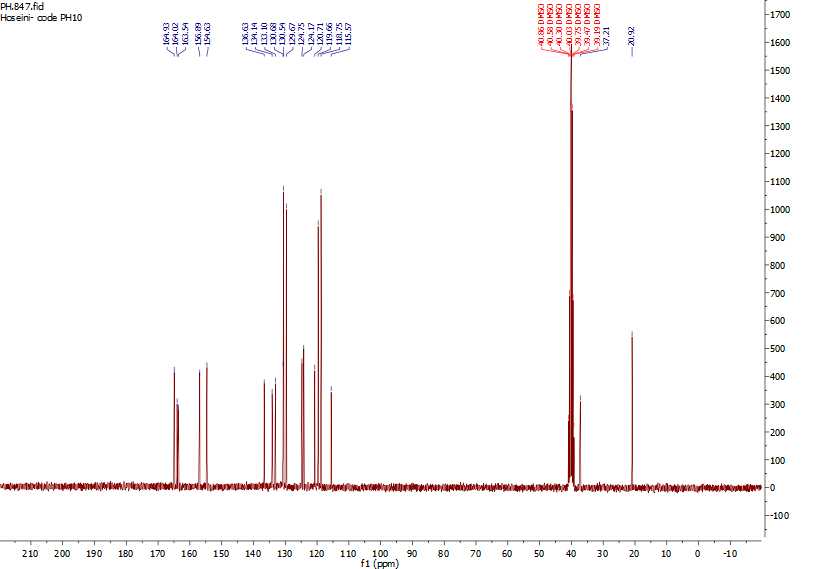
*

*N-(4-methoxyphenyl)-2-((5-(2-phenoxyphenyl)-1,3,4-oxadiazol-2-yl)thio)acetamide (****8c****)*

*
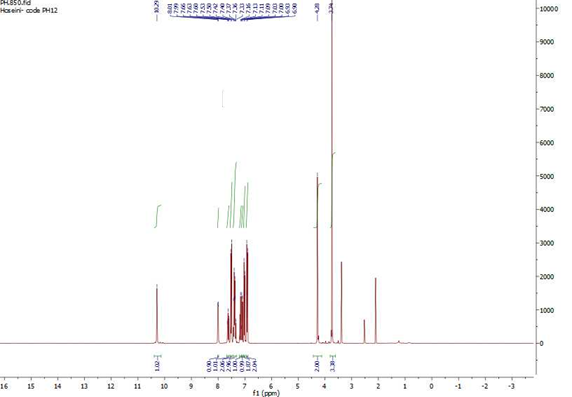
*

*
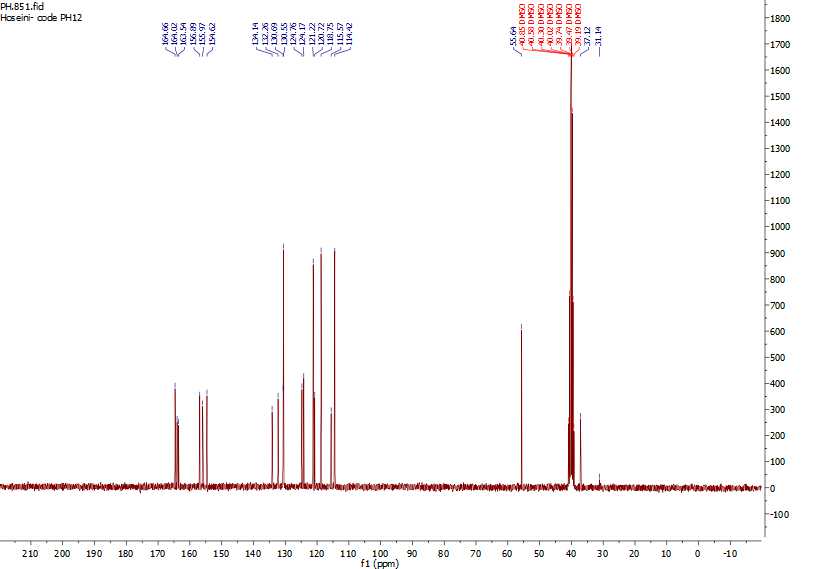
*

*N-(4-fluorophenyl)-2-((5-(2-phenoxyphenyl)-1,3,4-oxadiazol-2-yl)thio)acetamide (****8d****)*

*
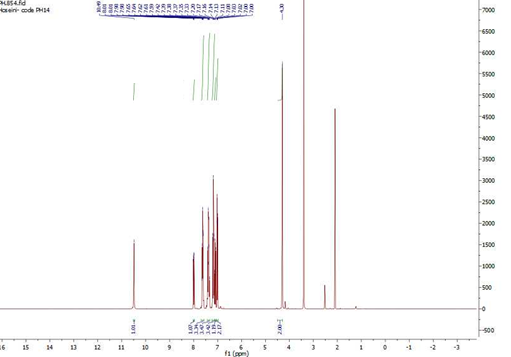
*

*
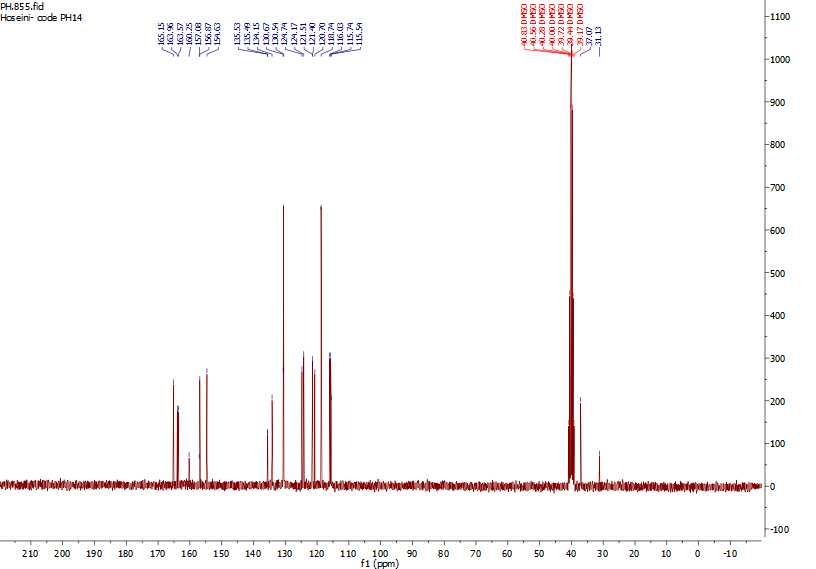
*

*N-(2,4-difluorophenyl)-2-((5-(2-phenoxyphenyl)-1,3,4-oxadiazol-2-yl)thio)acetamide (****8e****)*

*
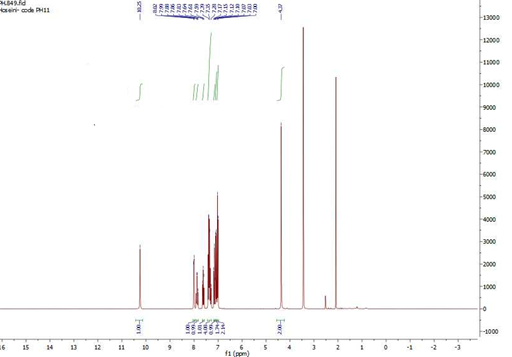
*

*
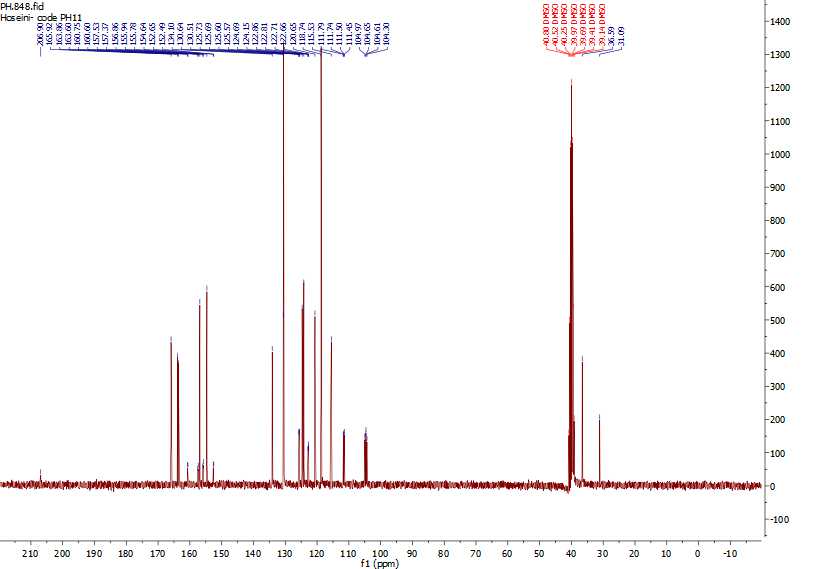
*

*N-(2-chlorophenyl)-2-((5-(2-phenoxyphenyl)-1,3,4-oxadiazol-2-yl)thio)acetamide (****8f****)*

*
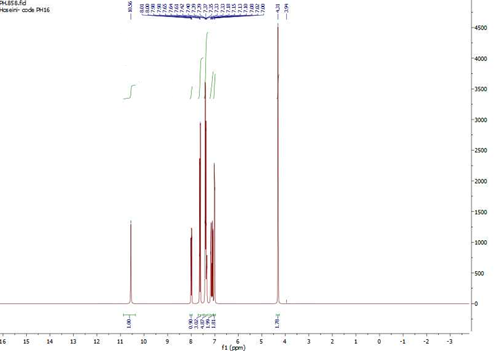
*

*
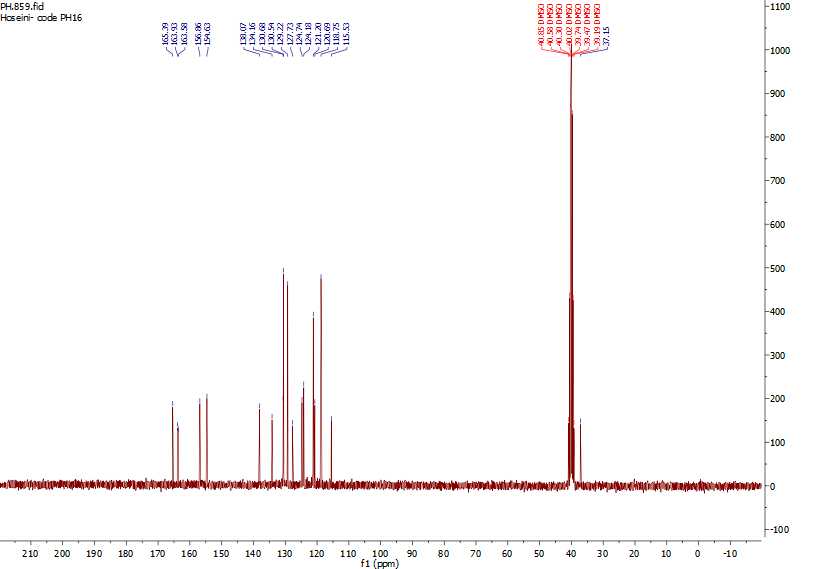
*

*N-(3-chlorophenyl)-2-((5-(2-phenoxyphenyl)-1,3,4-oxadiazol-2-yl)thio)acetamide (****8g****)*

*
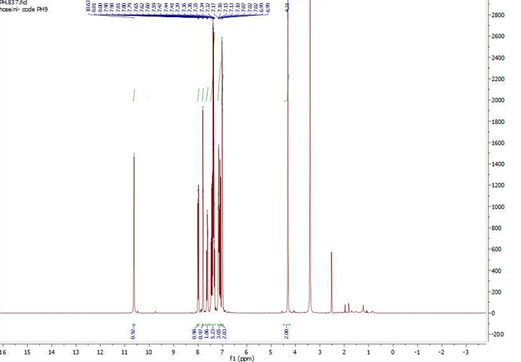
*

*
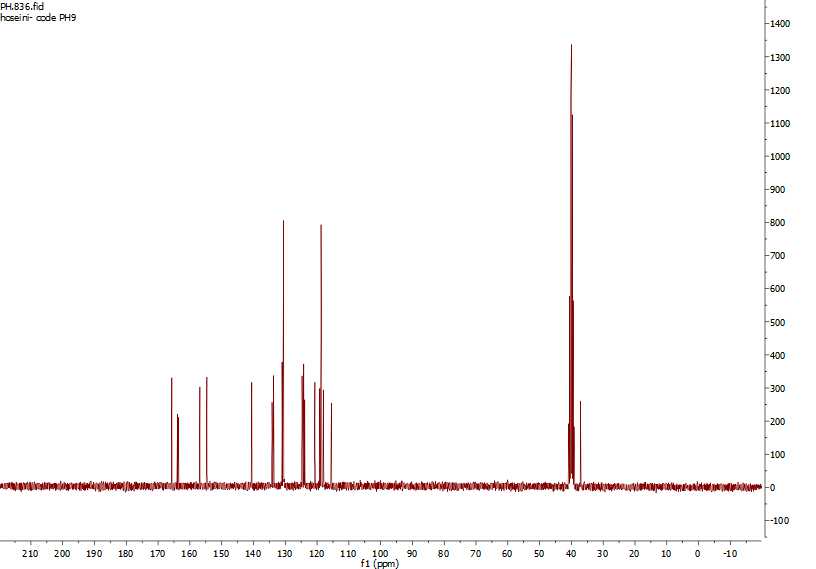
*

*N-(3,5-dichlorophenyl)-2-((5-(2-phenoxyphenyl)-1,3,4-oxadiazol-2-yl)thio)acetamide (****8h****)*

*
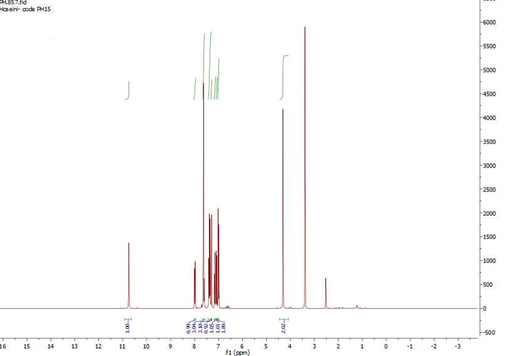
*

*
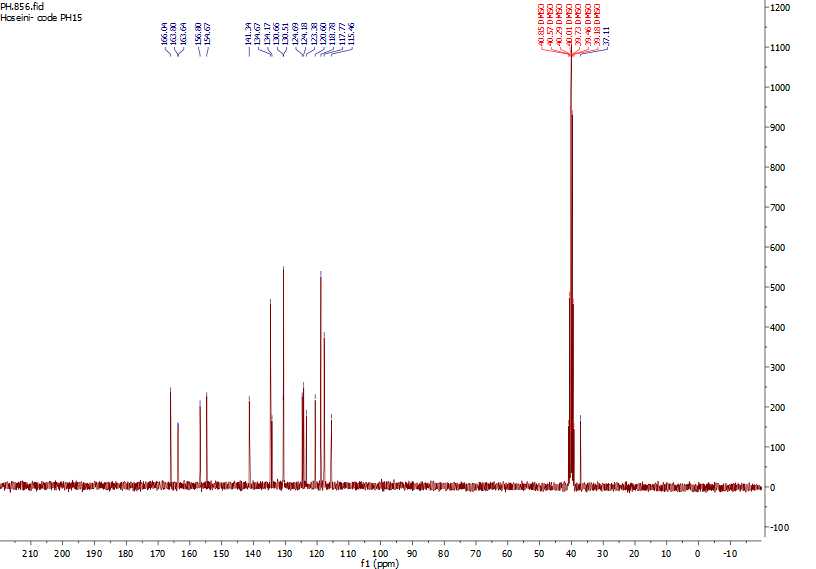
*

*N-(3-bromophenyl)-2-((5-(2-phenoxyphenyl)-1,3,4-oxadiazol-2-yl)thio)acetamide (****8i****)*

*
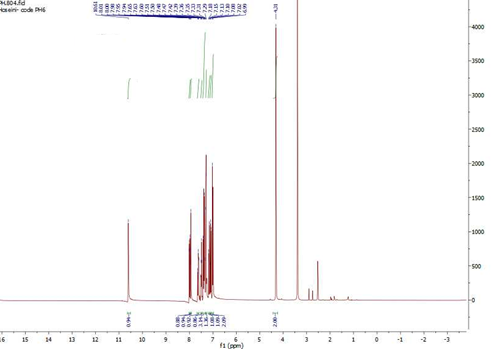
*

*
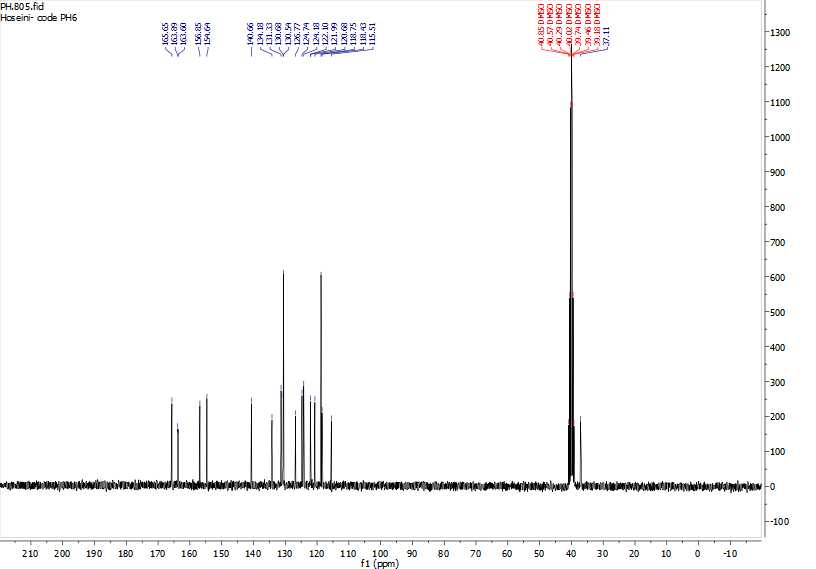
*

*N-(4-bromophenyl)-2-((5-(2-phenoxyphenyl)-1,3,4-oxadiazol-2-yl)thio)acetamide (****8j****)*

*
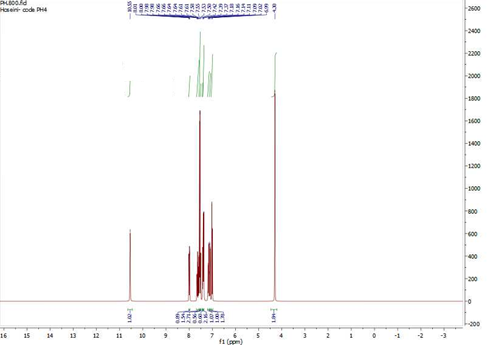
*

*
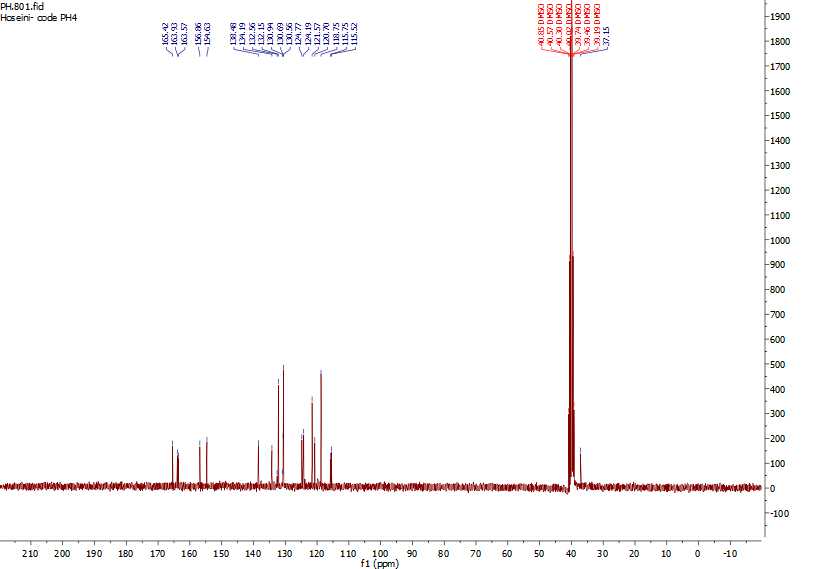
*

*N-(2-nitrophenyl)-2-((5-(2-phenoxyphenyl)-1,3,4-oxadiazol-2-yl)thio)acetamide (****8k****)*

*
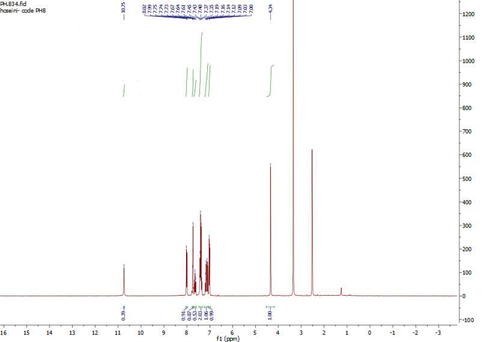
*

*
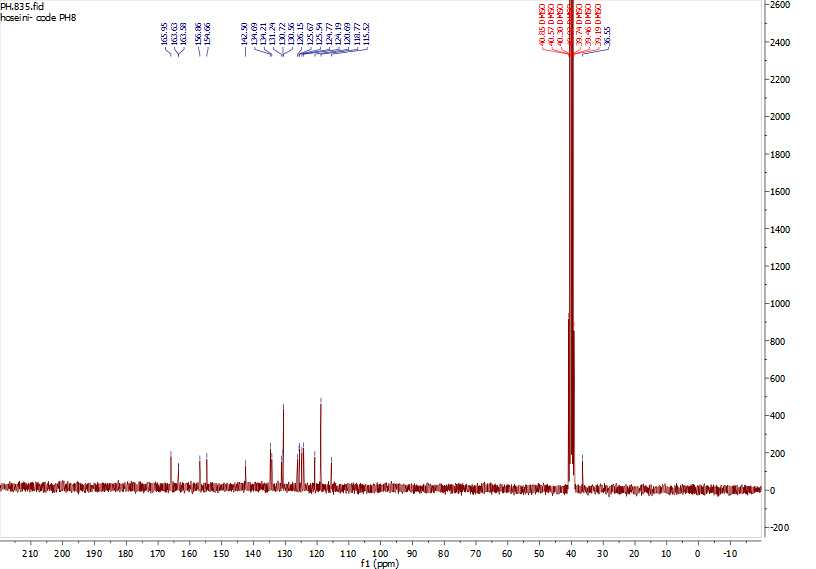
*

*N-(4-nitrophenyl)-2-((5-(2-phenoxyphenyl)-1,3,4-oxadiazol-2-yl)thio)acetamide (****8l****)*

*^
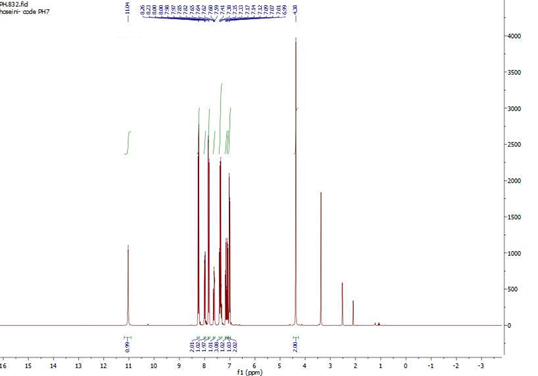
^*

*^
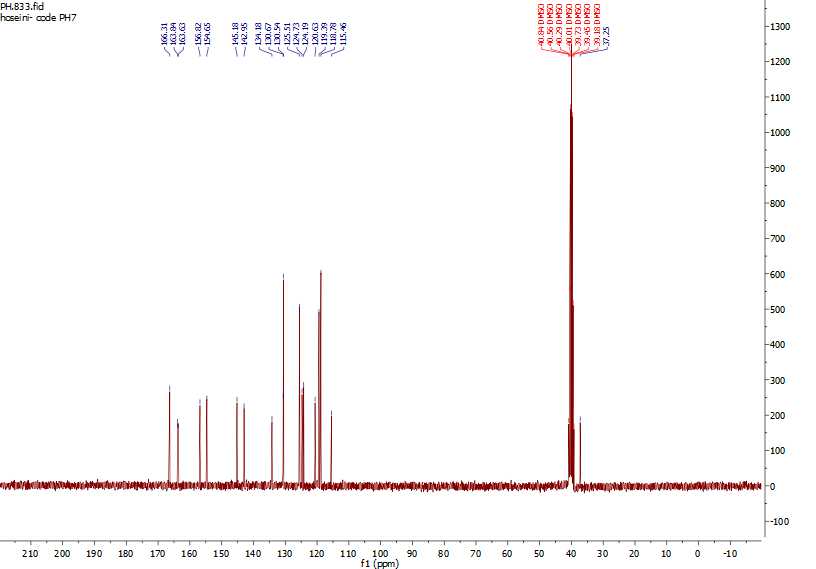
^*

*N-(2-methyl-3-nitrophenyl)-2-((5-(2-phenoxyphenyl)-1,3,4-oxadiazol-2-yl)thio)acetamide (****8m****)*

*
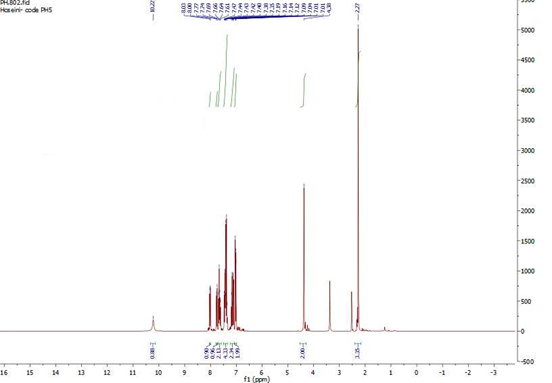
*

*
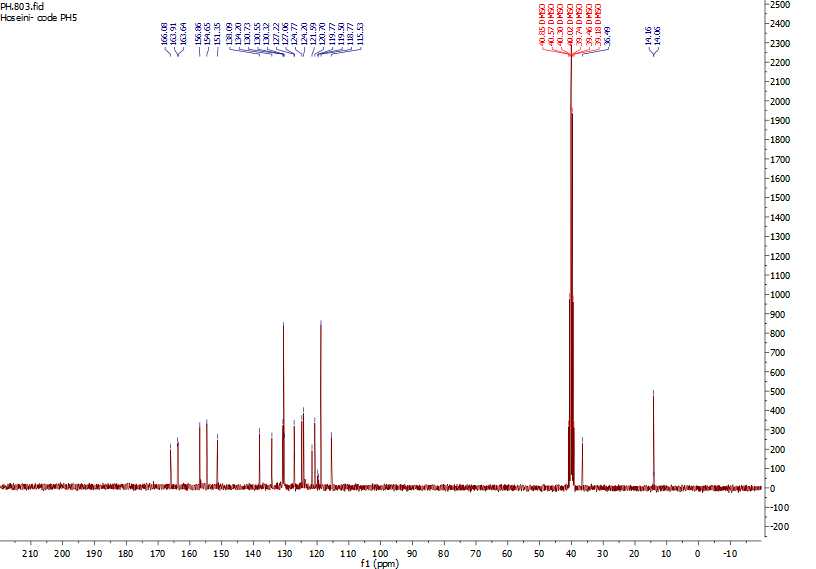
*
